# Genetic and Biochemical Characterization of AXC-2 from *Achromobacter ruhlandii*

**DOI:** 10.3390/pathogens13020115

**Published:** 2024-01-27

**Authors:** Mariana Papalia, Francisco González-Espinosa, Fátima Quiroga Castedo, Gabriel Gutkind, María Soledad Ramírez, Pablo Power, Marcela Radice

**Affiliations:** 1 Laboratorio de Resistencia Bacteriana, Instituto de Bacteriología y Virología Molecular, Facultad de Farmacia y Bioquímica, Universidad de Buenos Aires, Junín 956, Buenos Aires 1113, Argentina; espyfran@gmail.com (F.G.-E.); fatimaqc90@gmail.com (F.Q.C.); ggutkind@ffyb.uba.ar (G.G.); ppower@ffyb.uba.ar (P.P.); mradice@ffyb.uba.ar (M.R.); 2 Consejo Nacional de Investigaciones Científicas y Técnicas (CONICET), Godoy Cruz 2290, Buenos Aires C1425FQB, Argentina; 3 Center for Applied Biotechnology Studies, Department of Biological Science, California State University Fullerton, Fullerton, CA 92831, USA; msramirez@fullerton.edu

**Keywords:** *Achromobacter* spp., *A. ruhlandii*, AXC, carbapenemase

## Abstract

*Achromobacter* spp. are intrinsically resistant to multiple antibiotics and can also acquire resistance to those commonly used for the treatment of respiratory infections, especially in patients with cystic fibrosis. The aim of this study was to perform the genetic and biochemical characterization of AXC-2 from *A. ruhlandii* and to analyze all available AXC variants. Steady-state kinetic parameters were determined on a purified AXC-2 enzyme. It exhibited higher catalytic efficiencies towards amino-penicillins and older cephalosporins, while carbapenems behaved as poor substrates. Phylogenetic analysis of all *bla*_AXC_ variants available in the NCBI was conducted. AXC was encoded in almost all *A. ruhlandii* genomes, whereas it was only found in 30% of *A. xylosoxidans*. AXC-1 was prevalent among *A. xylosoxidans*. AXC variants were clustered in two main groups, correlating with the *Achromobacter* species. No association could be established between the presence of *bla*_AXC_ variants and a specific lineage of *A. xylosoxidans*; however, a proportion of AXC-1-producing isolates corresponded to ST 182 and ST 447. In conclusion, this study provides valuable insights into the genetic context and kinetic properties of AXC-2, identified in *A. ruhlandii*. It also provides a thorough description of all AXC variants and their association with *Achromobacter* species and various lineages.

## 1. Introduction

*Achromobacter* spp. are rod-shaped, aerobic, non-fermenting Gram-negative bacteria widely distributed in the environment and typically regarded as opportunistic pathogens.

The *Achromobacter* genus currently comprises 21 officially designated species, including *A. xylosoxidans* (the type species of the genus), *A. denitrificans*, *A. insolitus*, *A. marplatensis*, *A. piechaudii*, *A. ruhlandii*, *A. spanius*, *A. dolens*, *A. insuavis*, *A. pulmonis*, *A. deleyi*, *A. mucicolens*, *A. aegrifaciens*, *A. animicus*, *A. anxifer*, *A. kerstersii*, *A. sediminum*, *A. agilis*, *A. aloeverae*, *A. pestifer*, *A. aestuarii*, and various genogroups [1].

*A. xylosoxidans* was the sole species when the *Achromobacter* genus was proposed by Yabuuchi et al. in 1981 [2]. *A. ruhlandii* and *A. piechaudii* were reassigned from the *Alcaligenes* genus by Yabuuchi and Kawamura et al. in 1998, while *A. xylosoxidans* subsp. *denitrificans* was reclassified as *A. denitrificans* [3]. Subsequently, *A. spanius*, *A. insolitus*, and *A. marplatensis* were described by Gomila et al. in 2011 [4]. More recently, the remaining taxa were incorporated into the genus [5].

The accurate species identification of *Achromobacter* isolates is challenging, and unfortunately, they are often erroneously identified as *A. xylosoxidans*. Traditional phenotypic identification schemes frequently fail to discriminate between the various species within the *Achromobacter* genus. In 2013, the amplification and sequencing of the conserved gene *nrd*A was proposed for identification [6]. This method, along with multilocus sequence typing (MLST), is currently widely employed in research laboratories [7]. Additionally, identification can also be conducted by calculating the average nucleotide identity (ANI) in genomes. Veschetti et al. established that isolates with ANI ≥ 95% are classified within the same species, while isolates with ANI < 95% are considered to belong to different genogroups when compared to all available genomes of *Achromobacter* species [8]. The authors also reported a noticeable correlation with both MLST analysis and sequencing of the *nrd*A gene. This method proves valuable when genomes are accessible. However, sequence-based identification techniques are not practical for clinical microbiology laboratories. More recently, mass spectrometry (MALDI-TOF) has been introduced as a valuable tool in microbiology laboratories, enabling rapid bacterial identification (30 min). While this technique generally facilitates the accurate identification of *A. xylosoxidans*, it may yield inconclusive or even erroneous results for less common species. Currently, several research groups are actively working to enhance databases and achieve reliable species-level identification within the *Achromobacter* genus [9,10].

*Achromobacter*, mainly *A. xylosoxidans*, has been associated with pneumonia, bacteremia, and even meningitis, predominantly in immunocompromised patients, although cases have also been reported in immunocompetent individuals [11]. Additionally, *Achromobacter* species, distinct from *A. xylosoxidans*, have traditionally been considered environmental inhabitants and have only recently been associated with human infections. *Achromobacter* spp. have been isolated from the blood cultures and cerebrospinal fluid of neonatal patients (in cases of pneumonia or meningitis) and are linked to high mortality rates [12]. The majority of infections are acquired in the healthcare setting, while only one-third are in the community.

Since the year 2000, *Achromobacter* spp. have been recognized as emerging pathogens, especially in patients with cystic fibrosis (CF), as their prevalence in respiratory secretions has shown an upward trend [13]. The reported prevalence rate for *A. xylosoxidans* in cystic fibrosis centers worldwide is approximately 10%, although this rate is steadily increasing, with reports of prevalence reaching up to 29% [14,15]. This increase is likely attributed to the prolonged life expectancy of patients with CF, the selective pressures exerted by multi-antimicrobial therapy, and advancements in microbiological and molecular techniques. *A. xylosoxidans* is the most frequent *Achromobacter* species reported from the respiratory samples in these patients. Moreover, *A. ruhlandii* stands as the second most prevalent species in the Americas [6,16], while *A. dolens* and *A. insuavis* exhibit a higher prevalence in Europe [17,18].

*Achromobacter* spp. represent a serious threat to patients, as members of this genus are intrinsically resistant to multiple antibiotics, including cephalothin, cefoxitin, cefotaxime, aztreonam, and aminoglycosides [19]. Furthermore, *Achromobacter* species can acquire resistance to antimicrobial agents commonly employed in the treatment of respiratory infections, such as colistin, carbapenems, and fluoroquinolones. Extended-spectrum β-lactams are often considered the primary therapeutic option for treating *Achromobacter* infections in patients with cystic fibrosis, while carbapenems are reserved as last-resort antibiotics. Inhaled antibiotics, such as colistin or tobramycin, have been demonstrated to provide benefits as adjuvants to intravenous medications [20].

The two main intrinsic resistance mechanisms in *Achromobacter* species include multidrug efflux pumps, and chromosomal OXA-114-like β-lactamases [19]. *Achromobacter* spp. harbor two well-characterized multidrug efflux pumps. The AxyABM efflux pump is present in all publicly available *Achromobacter* genomes and shares common properties with the MexAB-OprM efflux pump of *P. aeruginosa* [21]. Several studies have demonstrated that imipenem is more effective than meropenem against *Achromobacter* isolates, possibly due to the overexpression of this efflux pump [22]. AxyABM plays a significant role in extruding aztreonam and cephalosporins other than cefepime and cefuroxime. The second efflux pump, AxyXY-OprZ, has a broader spectrum and is involved in extruding aminoglycosides, cefepime, carbapenems, fluoroquinolones, tetracyclines, and erythromycin [23,24,25]. AxyXY-OprZ is the primary determinant of high-level intrinsic aminoglycoside resistance in *Achromobacter* spp. For other antibiotics, AxyXY-OprZ appears to contribute to resistance, rather than being the primary resistance mechanism [19].

Regarding β-lactamases, *A. xylososidans* produces a constitutive chromosomal β-lactamase known as OXA-114, exhibiting activity against penicillin G, early cephalosporins, piperacillin, and ticarcillin [26]. However, extended-spectrum cephalosporins such as ceftazidime, cefotaxime, and cefepime are not substrates for OXA-114-like enzymes. OXA-114 demonstrates poor hydrolytic activity against imipenem. Additionally, other OXA-type enzymes were identified, proving to be ubiquitous and species-specific, such as OXA-258 in *A. ruhlandii*, OXA-243 in *A. insuavis*, and OXA-364 in *A. dolens* [27,28]. These enzymes efficiently hydrolyze penicillins and first-generation cephalosporins, but not third-generation cephalosporins and carbapenems.

Acquired antimicrobial resistance can occur through mutational events, alteration in gene expression, or acquisition of resistance markers encoded within mobile genetic elements. In terms of β-lactam resistance, plasmid-encoded enzymes have sporadically been reported in *A. xylosoxidans*, including broad-spectrum β-lactamases (BSBLs) and extended-spectrum β-lactamases (ESBLs), such as VEB-1 [29]. Concerning carbapenem resistance, the presence of class B enzymes (IMP-1, IMP-10, VIM-1, and VIM-2) has also been documented [30,31,32,33]. More recently, in 2018, a novel class A β-lactamase named AXC-1 (*A. xylosoxidans* carbapenemase), exhibiting slight activity against meropenem, was reported in *A. xylosoxidans* [34]. Kinetics parameters for this enzyme have not been described. Furthermore, in 2023, *bla*_AMZ–1_ was identified and characterized in *Achromobacter mucicolens*, demonstrating resistance against certain β-lactam antibiotics, including amoxicillin and cephalothin [35].

Although studies concerning *A. ruhlandii* antimicrobial resistance are limited, it has been reported that this species may exhibit a more resistant phenotype. A pandrug-resistant *A. ruhlandii* epidemic clone designated the Danish epidemic strain (DES) spread among patients with CF in 2006 in two Danish cystic fibrosis centers [36,37].

In a previous study, we characterized *Achromobacter* isolates recovered from respiratory samples of patients with CF [38]. Based on phenotypic and molecular techniques, *A. xylosoxidans* was the most frequently identified *Achromobacter* species in the airways of these patients, followed by *A. ruhlandii* as the second most prevalent. *Achromobacter* isolates included in this study were resistant to a wide range of antibiotics, including fluoroquinolones, aminoglycosides, and most broad-spectrum β-lactams. While carbapenems proved to be the most effective antibiotics, it is noteworthy that 3 out of 7 *A. ruhlandii* isolates and 1 out of 26 *A. xylosoxidans* isolates were resistant [38].

Considering the limited knowledge regarding *A. ruhlandii* and its antimicrobial resistance, efforts were focused on characterizing these isolates. Whole-genome sequencing was performed on one of the carbapenem-resistant *A. ruhlandii* isolates mentioned above. This particular isolate, Ar38, was recovered from the sputum of a pediatric patient in Buenos Aires in 2007 (ASM280300v1). In-depth in silico genome analysis revealed the presence of three β-lactamase coding genes, including a new *bla*_AXC_ variant.

The aim of this study was to conduct genetic and biochemical characterization of this AXC variant from *A. ruhlandii* Ar38 and to evaluate its possible contribution to the observed resistance phenotype. Additionally, a phylogenetic analysis of all available AXC variants and their presence in different *Achromobacter* species was investigated.

## 2. Materials and Methods

### 2.1. Genetic and Biochemical Characterization of AXC-2 from Ar38

#### 2.1.1. Whole-Genome Sequencing of *A. ruhlandii* Ar38

Whole-genome sequencing (WGS) was conducted using Illumina GAIIx, with Nextera XT libraries utilized for sample preparation. De novo assembly was performed with the SPAdes assembler v. 3.10.1 [39]. The RAST server was used to predict all open reading frames and their genetic context [40]. Sequence alignment and similarity searching to confirm predictions were accomplished using BLAST software v. 2.15.0.

#### 2.1.2. Comparative Analysis of AXC Sequences Deposited in the NCBI Database

Sequences of the 7 AXC variants deposited to date were downloaded. Alignment was performed using ClustalX. Analysis of the genetic context of these *bla*_AXC_ was performed using RAST and BLAST tools v. 2.15.0.

#### 2.1.3. β-Lactamase Production and Purification

Full-length *bla*_AXC-2_ was amplified by PCR using custom-designed primers AXC-F (5′ CCATATGTTGCTGACGAGAAGAAGAACCTT 3′), containing the *Nde*I restriction site, and AXC-R (5′ TCGAATTCCTAGCCCAATGCCGCCA 3′), containing the *Eco*RI restriction site. The *Nde*I-*EcoR*I amplicon was cloned into the pK19 vector and the pET-28a(+) expression vector (Novagen, Inc., Madison, WI, USA). The authenticity of the cloned fragments was confirmed by sequencing.

*Escherichia coli* TOP 10F’ was transformed with the pK19-AXC-2 recombinant plasmid, and the minimum inhibitory concentrations (MICs) of a representative panel of β-lactam antibiotics were determined using the broth microdilution test, according to CLSI guidelines.

A pET-AXC-2 recombinant plasmid was introduced into *E. coli* BL21 (DE3) (Novagen Inc., Madison, WI, USA) to overproduce the enzyme. An overnight culture of *E. coli* BL21(DE3)-pET-AXC-2 was diluted (1/20) in 1 L LB medium containing 30 µg/mL kanamycin (Sigma-Aldrich, Misuri, USA) and grown at 37 °C until 0.8 OD units (λ = 600 nm). IPTG (Sigma-Aldrich, Misuri, USA) was added at a final concentration of 1 mM, and culture was grown at 18 °C overnight. After centrifugation at 8000× *g* at 4 °C, cells were resuspended in 25 mL of 25 mM sodium phosphate buffer (pH 7.5). Cells were disrupted by sonication, and cellular debris was separated by centrifugation. The supernatant fraction of the induced culture displayed the highest specific activities when measured spectrophotometrically, with total enzymatic extracts and 100 μM cephalothin as a substrate.

Enzyme purification was carried out as follows. The supernatant fraction was dialyzed overnight at 4 °C against the same buffer used to equilibrate the ion-exchange columns (25 mM sodium phosphate buffer pH 6.5; buffer A) and clarified by centrifugation at 18,000× *g* for 30 min and filtration through a 0.45 μm pore membrane. Then, it was loaded onto a 5 mL Hi Trap^TM^ SP HP column (flow rate, 1 mL/min) (GE Healthcare Life Sciences, Milwaukee, WI, USA) equilibrated with buffer A. The column was thoroughly washed to remove unbound proteins, and the β-lactamase was eluted with a linear gradient of 1 M sodium chloride. β-lactamase containing fractions were pooled, and a second purification was performed. For this purpose, an ion exchange was carried out using a 1 mL RESOURCE^TM^ S column (GE Healthcare Life Sciences, Milwaukee, WI, USA) equilibrated with buffer A. Once again, the column was washed to remove unbound proteins, and the β-lactamase was eluted with a linear gradient of 1 M sodium chloride. β-lactamase containing fractions were pooled and dialyzed overnight against 25 mM sodium phosphate buffer (pH 7.5). Purified AXC-2 was stored at −20 °C until use. Enzyme purity was over 99%, as estimated by SDS-PAGE 

#### 2.1.4. Kinetic Studies

Steady-state kinetic parameters (*k*_cat_ and *K_m_*) were determined by measuring the rate of hydrolysis of different substrates, using an ultraviolet/visible (UV/Vis) spectrophotometer [41]. The reactions were performed at 25 °C in 25 mM sodium phosphate buffer (pH 7.5). The *k*_cat_ and *K_m_* values were determined for a representative set of β-lactam antibiotics, including ampicillin, penicillin, cephalexin, cefoxitin, ceftazidime, ceftriaxone, cefepime, imipenem, and meropenem.

### 2.2. In Silico Analysis of AXC Variants from Achromobacter *spp.*

#### 2.2.1. Phylogenetic Analysis of AXC Variants from *Achromobacter* Genomes Available in the Genome NCBI Database

A search for *bla*_AXC_ variants was conducted using BlastN v. 2.15.0. Subsequently, all available genomes of *Achromobacter* species where *bla*_AXC_ was detected were downloaded. Species identity was confirmed by searching for the *nrd*A gene and comparing alleles according to the PubMLST database (https://pubmlst.org/organisms/Achromobacter-spp. accessed on 10 November 2023). Genomes that displayed low-quality or small-length sequences were not included. The alignment of AXC variants translated from the aforementioned genomes was performed using ClustalX. A dendrogram based on maximum-likelihood estimation was conducted using MEGA 5.05 software.

#### 2.2.2. Analysis of the Distribution of AXC in *A. xylosoxidans* Genomes

To highlight the distribution of AXC in *A. xylosoxidans*, a phylogenetic analysis was performed. PROKKA was used for genome annotation; Roary for phylogenetic analysis based on core genes, using a blastp 80% similarity threshold; and the SNPsites program for core SNP filtering. The IQ-TREE plus Microreact was employed for the construction and visualization of the phylogenetic tree.

#### 2.2.3. Analysis of the Genetic Context of *bla*_AXC_ in *A. xylosoxindans* and *A. ruhlandii* Genomes

*bla*_AXC_ genetic context was investigated in selected *A. xylosoxidans* and *A. ruhlandii* genomes downloaded from the NCBI genome database. Clinker was used for the alignment and graphical representation of the genetic context.

## 3. Results

Three putative β-lactamase coding genes were detected by WGS and confirmed through in silico genomic analysis in Ar38. One of these open reading frames (ORFs) corresponded to *bla*_OXA-258_ (PHIK01000006.1:64095-64919), previously characterized by our group and proposed as a species-specific gene for *A. ruhlandii* [42]. The second ORF corresponded to *bla*_AXC_, not previously reported in *A. ruhlandii,* and is the main focus of this study (PHIK01000028.1:44673-45569). The third ORF encoded a putative class C β-lactamase that remains uncharacterized (PHIK01000013.1:c178058-176736).

**Figure 1:**
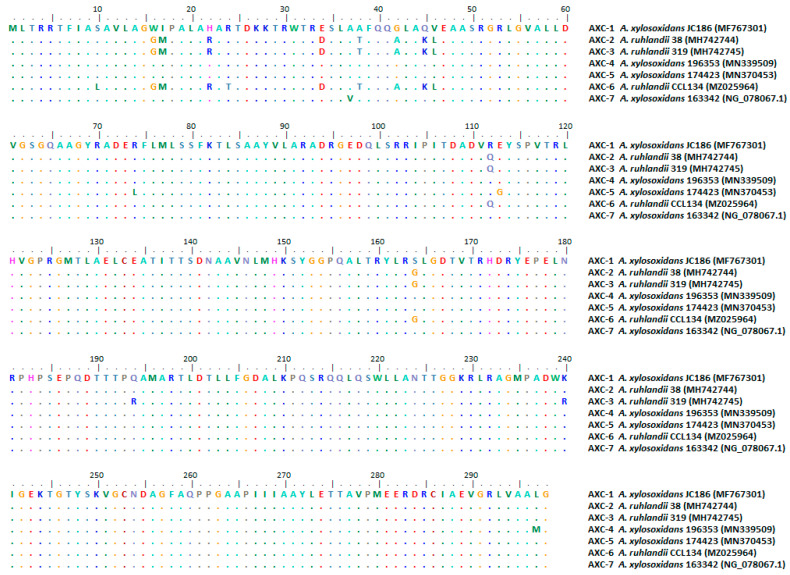
Alignment of AXC β-lactamases family.

AXC from Ar38 displayed 97% amino acid identity with AXC-1 from *A. xylosoxidans*, corresponding to a new variant named AXC-2, deposited in the GenBank database under the accession number MH742744. The alignment of AXC-2 and other AXC variants deposited in the NCBI database is shown in Figure 1.

**Figure 2:**
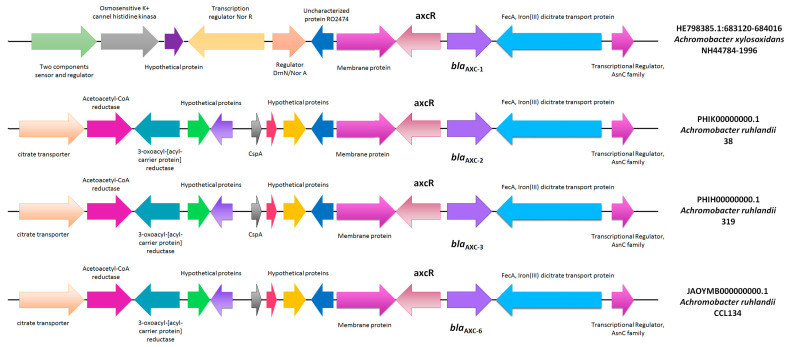
Genetic context of *bla*_AXC_ genes. Arrows represent the direction of transcription.

The presence of the transcriptional regulator *axcR*, previously described by Fleurbaaij et al. [34], was observed in the closest surrounding region of *bla*_AXC-2_, displaying 90% nucleotide similarity between Ar38 and the *A. xylosoxidans* strain previously described. The genetic context of the *bla*_AXC_ variants deposited in the NCBI was analyzed (Figure 2). It was observed that *bla*_AXC-2_, *bla*_AXC-3_, and *bla*_AXC-6_, present in *A. ruhlandii*, shared a common genetic environment, whereas *bla*_AXC-1_, present in *A. xylosoxidans*, displayed differences upstream of the gene. Unfortunately, a comparison of the genetic contexts for *bla*_AXC-4_, *bla*_AXC-5_, and *bla*_AXC-7_ could not be conducted due to the unavailability of the respective genomes. No insertion sequences or transposable elements that could indicate mobilization within the bacterial chromosome were observed.

An increase in the MIC values of ampicillin and cephalexin was observed in *E. coli* transformants producing AXC-2. Additionally, a slight increase in the MICs was observed for the carbapenems, although not enough to go over the breakpoints for these antibiotics (Table 1).

The purity of the AXC-2 extract was estimated to be >99% by sodium dodecyl sulfate-polyacrylamide gel electrophoresis. Kinetic constants for β-lactam antibiotics are shown in Table 2. It was observed that this enzyme exhibits higher catalytic efficiencies (*k*_cat_/ *K_m_*) towards amino-penicillins and older cephalosporins, and, to a lesser extent, benzyl-penicillin and ceftriaxone. Also, AXC-2 showed very low affinity for ceftazidime and cefoxitin, with *K*_m_ values in the millimolar range and not detectable by hydrolysis. Finally, cefepime and carbapenems behaved as poor substrates, mainly due to very low turnover (*k*_cat_) values. The slight hydrolysis of carbapenems by AXC-2 was consistent with the hydrolysis determined in the crude extracts obtained from the clinical isolate. This result suggested that this β-lactamase could be expressed in the isolate, even at a low level.

The presence of *bla*_AXC_ was detected in *A. ruhlandii, A. xylosoxidans*, and one *A*. *denitrificans* genome, but not in other species of this genus. Subsequently, all available *A. ruhlandii*, *A. xylosoxidans*, and *A. denitrificans* genomes were downloaded. Given the complexity associated with species identification within the genus and the potential misplacement of genomes in databases, their identity was confirmed by searching for the *nrd*A gene and comparing alleles according to the PubMLST database (https://pubmlst.org/organisms/Achromobacter-spp accessed on 10 November 2023). Of them, five isolates previously designated as *A. xylosoxidans* and one *A. denitrificans* corresponded to *A. ruhlandii.* Finally, a total of 144 *A. xylosoxidans* and 33 *A. ruhlandii* genomes were analyzed (Table S1, Supplementary Material).

Among the 33 *A. ruhlandii* genomes, *bla*_AXC_ was detected in 31, corresponding to AXC-2, AXC-3, AXC-6, and new AXC variants (Table S1, Supplementary Material). In a thorough examination of the two *A. ruhlandii* genomes lacking *bla*_AXC_, and its associated regulator, a uniform genetic context was revealed between them. These genomes exhibited consistent *nrd*A alleles and corresponded to ST 35. Additionally, a putative transposase, InsK, was detected in association with the insertion sequence element IS*150*, in the surrounding region of the *bla*_AXC_ location observed in other *A. ruhlandii* genomes. These collective findings give rise to a credible hypothesis of a gene loss event within this species.

Of the 144 *A. xylosoxidans* genomes, 40 were found harboring a *bla*_AXC_ gene. Comparison of *A. xylosoxidans* genomes through core gene analysis is available at https://microreact.org/project/knWPR2m1ErkcrWDFyVyabh-arbol-axylo-con-referencias-de-araiad. (accessed on 10 November 2023) No association was observed between the presence of AXC, or even its variants, and any specific ST; however, a discernible trend indicates that a proportion of those *A. xylosoxidans* carrying *bla*_AXC_ corresponded to ST 182 and 447.

**Figure 3:**
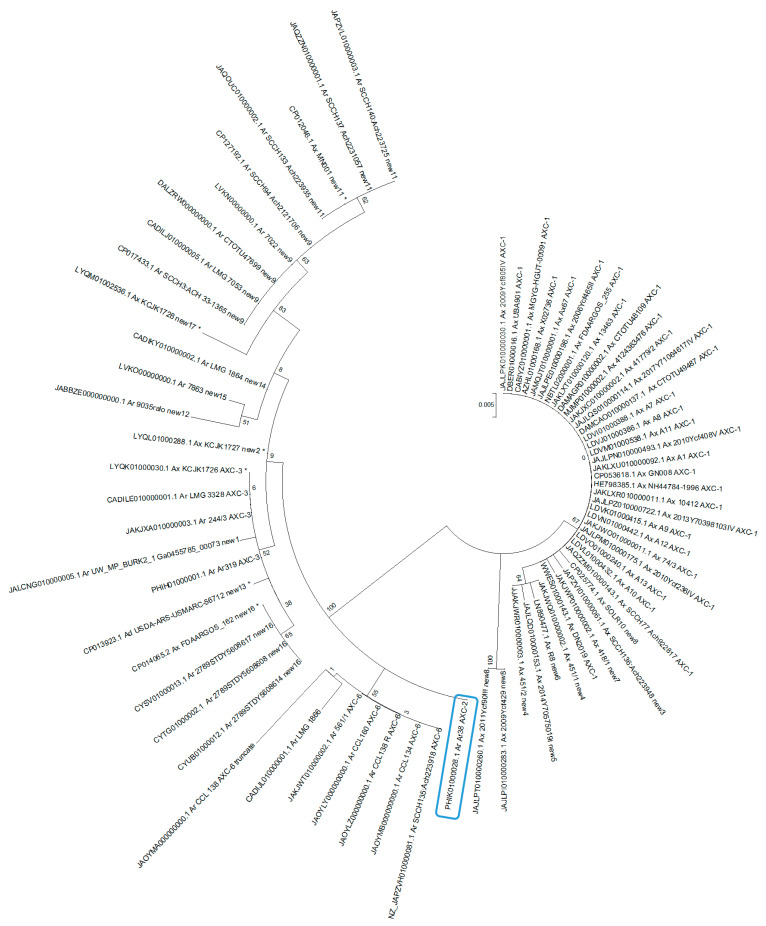
Dendrogram based on the maximum-likelihood estimation conducted using MEGA 5.05 for AXC genes. The enzyme AXC from Ar38 is highlighted in blue. * Genome analysis and organism identification by *nrd*A confirms that these isolates belong to the species *A. ruhlandii* and have been inaccurately identified.

A dendrogram of all AXC variants downloaded from *Achromobacter* genomes revealed that these enzymes clustered into two main groups. One group corresponded to those variants detected in *A. ruhlandii*, while the other clustered those from *A. xylosoxidans*. (Figure 3; and Table S1, Supplementary Material).

Analysis of the genetic context of *bla*_AXC_ in selected *A. ruhlandii* and *A. xylosoxidans* genomes (Figure S2, Supplementary Material) exhibited an overall conservative genetic context among isolates of the same species.

## 4. Discussion

This study represents the first comprehensive biochemical and kinetic characterization of an AXC enzyme. AXC-1 was previously referred to as a carbapenemase (*Achromobacter xylosoxidans* Carbapenemases); however, no kinetic data of this enzyme or any AXC variant is available [34]. AXC-2 displayed limited hydrolytic activity towards carbapenems; thus, the concomitant presence of multiple resistance mechanisms may be responsible for the carbapenem resistance observed in *A. ruhlandii* Ar38. Upon analyzing the genetic context of *bla*_AXC-2_, the presence of a regulator, *axcR,* was detected. AxcR exhibited 94% identity with the previously reported AxcR of *A. xylosoxidans* [34]. Mutations in the regulator could be responsible for changes in enzyme expression. Considering the presence of a regulator, a plausible scenario similar to that observed in AmpC-type enzymes can be hypothesized [43]. In isolates harboring an inducible *bla*_AmpC_ gene, its expression is determined by the regulator. In some cases, it might be stably derepressed, either partially or entirely, due to mutations in *ampR*, leading to the sustained production of large quantities of the enzyme. Additional experiments are essential to confirm this hypothesis in AXC.

Moreover, as mentioned by Chalhoub et al. [44], the active efflux of antibiotics in *A. xylosoxidans* mediated by the AxyABM, AxyXY-OprZ, or AxyEF-OprN pumps is potentially involved in antibiotic resistance. In a previous study, we characterized the AxyABM pump from *A. ruhlandii* Ar38, proving its active transport capability for chloramphenicol, nalidixic acid, and trimethoprim/sulfamethoxazole, while showing no efflux activity towards carbapenems [27]. Furthermore, we detected the presence of AxyXY-OprZ coding genes in *A ruhlandii* Ar38 genome, which has been proposed by multiple authors as a potential mediator of carbapenem efflux [23,24]. Further studies will be conducted to evaluate the carbapenem extrusion capability of this pump.

Currently, seven AXC variants have been documented in the NCBI database. However, BlastN-based analysis for *bla*_AXC_ identified many new enzymatic variants. Notably, *bla*_AXC_ was only detected in *A. ruhlandii* and *A. xylosoxidans* genomes, but not in any other bacterial species. An AXC enzyme was encoded in almost all available *A. ruhlandii* genomes, whereas it was only found in 40 out of 144 *A. xylosoxidans*. The AXC-1 coding gene was prevalent among the *A. xylosoxidans* isolates.

The phylogenetic analysis of AXC variants revealed two main clusters, indicating a correlation between AXC variants and *Achromobacter* species. When analyzing *A. xylosoxidans* genomes, no association could be established between the presence of *bla*_AXC_, or its variants, and a specific lineage. However, a noticeable pattern suggests that a proportion of AXC-1-producing *A. xylosoxidans* corresponded to ST 182 and 447. Interestingly, ST 182 isolates were recovered from both patients with CF and patients with non-CF in Thailand, Russia, and France, and a multidrug-resistant isolate in the United States was also found [45]. On the other hand, ST 447 was described in France in isolates recovered from patients with CF, and was associated with acquired resistance to ciprofloxacin [46].

The genetic context remained consistent among all variants observed in *A. ruhlandii*, with no discernible insertion sequences or transposable elements that might suggest mobilization within the bacterial chromosome. In the two *A. ruhlandii* genomes lacking *bla*_AXC_, its associated regulator was also absent; however, a putative transposase, InsK, was identified in the proximity of this region, probably indicative of a gene loss event. For its part, the genetic context of *bla*_AXC_ in *A. xylosoxidans* was similar in all analyzed genomes but differed from that observed for *A. ruhlandii.*

In conclusion, this study provides valuable insights into the genetic context and kinetic properties of AXC-2 identified in *A. ruhlandii*. AXC-2 displayed limited hydrolytic activity against carbapenems, prompting the need for future studies to evaluate the role of *axcR* in the expression of *bla*_AXC-2_, and the contribution of the AxyXY-OprZ efflux pump. Furthermore, we provide a thorough description of all AXC variants and their association with *Achromobacter* species and their various lineages.

Understanding the clinical relevance of *A. ruhlandii,* mainly in patients with CF, and the resistance mechanisms is of utmost importance for making accurate decisions in antimicrobial treatment. A study conducted in Brazil revealed that over half (57.1%) of the patients from whom *A. ruhlandii* was isolated developed a chronic infection, whereas the rate was only 13.3% for those with *A. xylosoxidans* [16]. These observations suggest that patients colonized/infected with *A. ruhlandii* are at a higher risk of developing chronic infection. Accordingly, in the study reported in Denmark, concerning the DES, 13 patients were chronically infected. Moreover, this species has been implicated in causing cross-infections among patients with cystic fibrosis, even in scenarios of limited and indirect contact, raising concerns within the CF communities [47]. Additionally, it has been reported that *A. ruhlandii* isolates present higher resistance levels compared to other species within the genus [36,37,38]. This knowledge is critical for optimizing the management of infections and ensuring the ongoing success of antibiotic therapies in the fight against infectious diseases.

## Figures and Tables

**Table 1:** β-lactams MICs for *A. ruhlandii* strain Ar38, *E. coli* TOP10F’ harboring pK19 plasmid, *E. coli* TOP10F’ harboring pK19 plasmid expressing AXC-2, and *E. coli* TOP10F’ (reference strain). The changes in MIC due to the presence of the β-lactamase can be observed in bold, in comparison with the control.

	MIC (μg/mL)								
	AMP	PIP	CEX	FOX	CAZ	CRO	FEP	IPM	MEM
*E. coli* TOP 10 F´	8	1	4	2	0.25	≤0.125	≤0.125	0.125	0.125
*E. coli* TOP 10 F´ + pK19	8	1	4	2	0.25	≤0.125	≤0.125	0.125	0.125
*E. coli* TOP 10 F´ + pK19 + AXC-2	**32**	2	**64**	2	0.5	≤0.125	≤0.125	**0.5**	**0.5**
*A. ruhlandii* Ar38	512	16	512	256	16	512	64	16	8

AMP: ampicillin, PIP: piperacillin, CEX: cephalexin, FOX: cefoxitin, CAZ: ceftazidime, CRO: ceftriaxone, FEP: cefepime, IPM: imipenem, MEM: meropenem.

**Table 2:** Steady-state kinetic parameters of AXC-2.

Antibiotic	*K_m_* (μM)	*k*_cat_ (s^−1^)	*k*_cat_/*K*_m_ (μM^−1^·s^−1^)
Nitrocefin	202 ± 24	40 ± 3	0.20 ± 0.04
Ampicillin	344 ± 14	62 ± 5	0.18 ± 0.02
Benzyl-penicillin	1297 ± 409	125 ± 28	0.10 ± 0.05
Cephalexin	74 ± 14	16 ± 2	0.22 ± 0.07
Cefoxitin	>1000	NH	ND
Ceftazidime	>1000	NH	ND
Ceftriaxone	59 ± 6	5.5 ± 0.3	0.09 ± 0.01
Cefepime	872 ± 254	0.29 ± 0.07	0.0003 ± 0.0001
Imipenem	6.3 ± 0.8	0.0364 ± 0.0009	0.0058 ± 0.0009
Meropenem	8 ± 1	0.051 ± 0.002	0.007 ± 0.001

ND, not determined; NH, no hydrolysis was observed under the used experimental conditions.

## Data Availability

The data presented in this study are available on request to the corresponding author.

## Supplementary Materials

The following supporting information can be downloaded at: https://www.mdpi.com/article/10.3390/pathogens13020115/s1, Figure S1: Alignment of AXC variants found in *A. ruhlandii* and *A. xylosoxidans* genomes; Figures S2: *bla*_AXC_ genetic context investigated in selected *A. xylosoxidans* and *A. ruhlandii* genomes downloaded from NCBI genome database. Table S1: Distribution of *bla*_AXC_ in *A. xylosoxidans* and *A. ruhlandii* Genomes
