# Supplementary material for: Genetic and Biochemical Characterization of AXC-2 from Achromobacter ruhlandii
Source: Pathogens. 2024 Jan 27;13(2):115. doi: 10.3390/pathogens13020115 (PMC10893412; doi:10.3390/pathogens13020115)

Figure S1: Alignment of AXC variants found in *A. ruhlandii* and *A. xylosoxidans* genomes

```
                  10        20        30        40        50
         ....|....|....|....|....|....|....|....|....|....|
MLTRRTFIASAVLAGWIPALAHARTDKKTRWTRESLAAFQQGLAQVEAAS  LDVO01000240.1
..................................................  LDVL01000432.1
..................................................  JAJLPM010000175
..................................................  JAKJWO010000011
..................................................  LDVN01000442.1
..................................................  LDVK01000415.1
..................................................  JAJLPZ010000722
..................................................  JAKLXR010000011
..................................................  HE798385.1
..................................................  CP053618.1
..................................................  JAKLXU010000092
.............T....................................  CP025774.1
..................................................  JAJLPN010000493
..................................................  LDVM01000538.1
..................................................  LDVJ01000386.1
..................................................  LDVI01000388.1
..................................................  JAPZVI010000061
..................................................  DAMCAO010000137
..................................................  JAJLQS010000114
..................................................  JAKJXC010000002
..................................................  MJMP01000002.1
..................................................  DAMAGR010000002
..................................................  JAKJWP010000002
..................................................  JAKLXT010000120
.P................T...............................  JAJLPI010000283
..................................................  NBTL02000001.1
..................................................  JAJLPE010000196
.P................T...............................  JAJLPT010000260
..................................................  JAMQJT010000001
...............GM....R...........D...T...A..KL....  JAKJXA010000003
..................................................  AZHL01000168.1
....................................V.............  JAJLQD010000153
....................................V.............  LN890477.1
....................................V.............  JAKJWR010000003
....................................V.............  JAKJWQ010000002
..................................................  CABIYZ010000001
..................................................  JAJLPK010000030
...............GM....R...........D...T...A..KL....  LYQL01000288.1
...............GM....R...........D...T...A..KL....  CP013923.1
...............GM....R...........D...T...A..KL....  CP014065.2
...............GM....R...........D...T...A..KL....  PHIK01000028.1
...............GM....R...........D...T...A..KL....  CYSV01000013.1
..................................................  DBER01000016.1
..................................................  JAQZZM010000143
...............GM....R...........D...T...A..KL....  CADILE010000001
.........L.....GM................D...T...S..KL....  JAQOUC010000002
.........L.....GM................D...T...S..KL....  LVKN00000000.1
.........L.....GM................D...T...S..KL....  DALZRW000000000
.........L.....GM................D...T...S..KL....  CP017433.1
.........L.....GM................D...T...S..KL....  CP012046.1
...............GM....R...........D...T...A..KL....  LYQK01000030.1
...............GM....R...........D...T...A..KL....  CYUB01000012.1
.........L.....GM....R.T.........D...T...A..KL....  JAOYLY000000000
...............GM.T..R...........D...T...A..KL....  JALCNG010000005
.........L.....GM................D...T...S..KL....  CADILJ010000005
...............GM....R...........D...T...A..KL....  CYTG01000002.1
...............GM....R...........D...T...A..KL....  PHIH01000001.1
.........L.....GM................D...T...S..KL....  JAQZZN010000001
.........L.....GM................D...T...S..KL....  CP127192.1
..............SGM.F..R...........D...T...A.VKL....  JABBZE000000000
```

```
..............SGM....R...........D...T...S..KL....   LVKO00000000.1
...............GM....R...........D...T...S..KL....   CADIKY010000002
..................................................   WWES01000143.1
.........L.....GM................D...T...S..KL....   LYQM01002536.1
.........L.....GM....R.T.........D...T...A..KL....   JAOYLZ000000000
.........L.....GM....R.T.........D...T...A..KL....   JAPZVH000000000
.........L.....GM....R.T.........D...T...A..KL....   JAOYMB000000000
.........L.....GM................D...T...S..KL....   JAPZVL010000003
.........L.....GM....R.T.........D...T...A..KL....   CADIJL010000001
.........L.....GM....R.T.........D...T...A..KL....   JAKJWT010000002
.........L.....GM....R.T.........D...T...A..KL....   JAOYMA000000000

         60        70        80        90        100
....|....|....|....|....|....|....|....|....|....|
RGRLGVALLDVGSGQAAGYRADERFLMLSSFKTLSAAYVLARADRGEDQL   LDVO01000240.1
..................................................   LDVL01000432.1
..................................................   JAJLPM010000175
..................................................   JAKJWO010000011
..................................................   LDVN01000442.1
..................................................   LDVK01000415.1
..................................................   JAJLPZ010000722
..................................................   JAKLXR010000011
..................................................   HE798385.1
..................................................   CP053618.1
..................................................   JAKLXU010000092
..................................................   CP025774.1
..................................................   JAJLPN010000493
..................................................   LDVM01000538.1
..................................................   LDVJ01000386.1
..................................................   LDVI01000388.1
..................................................   JAPZVI010000061
..................................................   DAMCAO010000137
..................................................   JAJLQS010000114
..................................................   JAKJXC010000002
..................................................   MJMP01000002.1
..................................................   DAMAGR010000002
..................................................   JAKJWP010000002
..................................................   JAKLXT010000120
......................Q...........................   JAJLPI010000283
..................................................   NBTL02000001.1
..................................................   JAJLPE010000196
......................Q...........................   JAJLPT010000260
..................................................   JAMQJT010000001
..................................................   JAKJXA010000003
..................................................   AZHL01000168.1
..................................................   JAJLQD010000153
..................................................   LN890477.1
..................................................   JAKJWR010000003
..................................................   JAKJWQ010000002
..................................................   CABIYZ010000001
..................................................   JAJLPK010000030
..................................................   LYQL01000288.1
..................................................   CP013923.1
..................................................   CP014065.2
..................................................   PHIK01000028.1
..................................................   CYSV01000013.1
..................................................   DBER01000016.1
..................................................   JAQZZM010000143
..................................................   CADILE010000001
..................................................   JAQOUC010000002
..................................................   LVKN00000000.1
..................................................   DALZRW000000000
```

```
.................................................. CP017433.1
.................................................. CP012046.1
.................................................. LYQK01000030.1
.................................................. CYUB01000012.1
.................................................. JAOYLY000000000
.................................................. JALCNG010000005
.................................................. CADILJ010000005
.................................................. CYTG01000002.1
.................................................. PHIH01000001.1
.................................................. JAQZZN010000001
.................................................. CP127192.1
.................................................. JABBZE000000000
.................................................. LVKO00000000.1
.................................................. CADIKY010000002
.................................................. WWES01000143.1
............G.R................................... LYQM01002536.1
.................................................. JAOYLZ000000000
.................................................. JAPZVH000000000
.................................................. JAOYMB000000000
.................................................. JAPZVL010000003
.................................................. CADIJL010000001
.................................................. JAKJWT010000002
.................................................. JAOYMA000000000

         110       120       130       140       150
....|....|....|....|....|....|....|....|....|....|
SRRIPITDADVREYSPVTRLHVGPRGMTLAELCEATITTSDNAAVNLMHK LDVO01000240.1
.................................................. LDVL01000432.1
.................................................. JAJLPM010000175
.................................................. JAKJWO010000011
.................................................. LDVN01000442.1
.................................................. LDVK01000415.1
.................................................. JAJLPZ010000722
.................................................. JAKLXR010000011
.................................................. HE798385.1
.................................................. CP053618.1
.................................................. JAKLXU010000092
.................................................. CP025774.1
.................................................. JAJLPN010000493
.................................................. LDVM01000538.1
.................................................. LDVJ01000386.1
.................................................. LDVI01000388.1
.................................................. JAPZVI010000061
.................................................. DAMCAO010000137
.................................................. JAJLQS010000114
.................................................. JAKJXC010000002
.................................................. MJMP01000002.1
.................................................. DAMAGR010000002
.................................................. JAKJWP010000002
.................................................. JAKLXT010000120
..........IQ...................................... JAJLPI010000283
.................................................. NBTL02000001.1
.................................................. JAJLPE010000196
..........IQ...................................... JAJLPT010000260
.................................................. JAMQJT010000001
...........Q...................................... JAKJXA010000003
.................................................. AZHL01000168.1
.................................................. JAJLQD010000153
.................................................. LN890477.1
.................................................. JAKJWR010000003
.................................................. JAKJWQ010000002
.................................................. CABIYZ010000001
```

```
..................................................  JAJLPK010000030
...........Q......................................  LYQL01000288.1
...........Q......................................  CP013923.1
...........Q......................................  CP014065.2
...........Q......................................  PHIK01000028.1
...........Q......................................  CYSV01000013.1
..................................................  DBER01000016.1
..................................................  JAQZZM010000143
...........Q......................................  CADILE010000001
...........QG.....................................  JAQOUC010000002
...........QG.....................................  LVKN00000000.1
...........QG.....................................  DALZRW000000000
...........QG.....................................  CP017433.1
...........QG.....................................  CP012046.1
...........Q......................................  LYQK01000030.1
...........Q......................................  CYUB01000012.1
...........Q......................................  JAOYLY000000000
...........Q......................................  JALCNG010000005
...........QG.....................................  CADILJ010000005
...........Q......................................  CYTG01000002.1
...........Q......................................  PHIH01000001.1
...........QG.....................................  JAQZZN010000001
...........QG.....................................  CP127192.1
...........Q......................................  JABBZE000000000
...........Q......................................  LVKO00000000.1
...........Q......................................  CADIKY010000002
..................................................  WWES01000143.1
...........Q..............L.......................  LYQM01002536.1
...........Q......................................  JAOYLZ000000000
...........Q......................................  JAPZVH000000000
...........Q......................................  JAOYMB000000000
...........QG.....................................  JAPZVL010000003
...........Q......................................  CADIJL010000001
...........Q......................................  JAKJWT010000002
...........Q......................................  JAOYMA000000000

         160       170       180       190       200
SYGGPQALTRYLRSLGDTVTRHDRYEPELNRPHPSEPQDTTTPQAMARTL  LDVO01000240.1
..................................................  LDVL01000432.1
..................................................  JAJLPM010000175
..................................................  JAKJWO010000011
..................................................  LDVN01000442.1
..................................................  LDVK01000415.1
..................................................  JAJLPZ010000722
..................................................  JAKLXR010000011
..................................................  HE798385.1
..................................................  CP053618.1
..................................................  JAKLXU010000092
..................................................  CP025774.1
..................................................  JAJLPN010000493
..................................................  LDVM01000538.1
..................................................  LDVJ01000386.1
..................................................  LDVI01000388.1
..................................................  JAPZVI010000061
..................................................  DAMCAO010000137
..................................................  JAJLQS010000114
..................................................  JAKJXC010000002
..................................................  MJMP01000002.1
..................................................  DAMAGR010000002
.......................H..........................  JAKJWP010000002
..................................................  JAKLXT010000120
```

```
. . . . . . . . . . . . . . . . . . I . . . . . . . . . . . . . . . . . . . . . . . . . . . . . .   JAJLPI010000283
. . . . . . . . . . . . . . . . . . . . . . . . . . . . . . . . . . . . . . . . . . . . . . . . . .   NBTL02000001.1
. . . . . . . . . . . . . . . . . . . . . . . . . . . . . . . . . . . . . . . . . . . . . . . . . .   JAJLPE010000196
. . . . . . . . . . . . . . . . . . I . . . . . . . . . . . . . . . . . . . . . . . . . . . . . .   JAJLPT010000260
. . . . . . . . . . . . . . . . . . . . . . . . . . . . . . . . . . . . . . . . . . . . . . . . . .   JAMQJT010000001
. . . . . . . . . . . . . G . . . . . . . . . . . . . . . . . . . . . . . . . . . . . R . . . . . .   JAKJXA010000003
. . . . . . . . . . . . . . . . . . . . . . . . . . . . . . . . . . . . . . . . . . . . . . . . . .   AZHL01000168.1
. . . . . . . . . . . . . . . . . . . . G . . . . . . . . . . . . . . . . . . . . . . . . . . . . .   JAJLQD010000153
. . . . . . . . . . . . . . . . . . . . . . . . . . . . . . . . . . . . . . . . . . . . . . . . . .   LN890477.1
. . . . . . . . . . . . . . . . . . . . . . . . . . . . . . . . . . . . . . . . . . . . . . . . . .   JAKJWR010000003
. . . . . . . . . . . . . . . . . . . . . . . . . . . . . . . . . . . . . . . . . . . . . . . . . .   JAKJWQ010000002
. . . . . . . . . . . . . . . . . . . . . . . . . . . . . . . . . . . . . . . . . . . . . . . . . .   CABIYZ010000001
. . . . . . . . . . . . . . . . . . . . . . . . . . . . . . . . . . . . . . . . . . . . . . . . . .   JAJLPK010000030
. . . . . . . . . . . . . G . . . . . . . . . . . . . . . . . . . . . . . . . . . . . R . . . . . .   LYQL01000288.1
. . . . . . . . . . . . . G . . . . . . . . . . . . . . . . . . . . . . . . . . . . . R . . . . . .   CP013923.1
. . . . . . . . . . . . . G . . . . . . . . . . . . . . . . . . . . . . . . . . . . . R . . . . . .   CP014065.2
. . . . . . . . . . . . . G . . . . . . . . . . . . . . . . . . . . . . . . . . . . . . . . . . . .   PHIK01000028.1
. . . . . . . . . . . . . G . . . . . . . . . . . . . . . . . . . . . . . . . . . . . R . . . . . .   CYSV01000013.1
. . . . . . . . . . . . . . . . . . . . . . . . . . . . . . . . . . . . . . . . . . . . . . . . . .   DBER01000016.1
. . . . . . . . . . . . . . . . . . . . . . . . . . . . . . . . . . . . . . . . . . . . . . . . . .   JAQZZM010000143
. . . . . . . . . . . . . G . . . . . . . . . . . . . . . . . . . . . . . . . . . . . R . . . . . .   CADILE010000001
. . . . . . . . . . . . . G . . . . . . . . . . . . L . . . . . . . . . . . . . . . . R . . . . . .   JAQOUC010000002
. . . . . . . . . . . . . G . . . . . . . . . . . . . . . . . . . . . . . . . . . . . R . . . . . .   LVKN00000000.1
. . . . . . . . . . . . . G . . . . . . . . . . . . . . . . . . . . . . . . . . . . . R . . . . . .   DALZRW000000000
. . . . . . . . . . . . . G . . . . . . . . . . . . . . . . . . . . . . . . . . . . . R . . . . . .   CP017433.1
. . . . . . . . . . . . . G . . . . . . . . . . . . L . . . . . . . . . . . . . . . . R . . . . . .   CP012046.1
. . . . . . . . . . . . . G . . . . . . . . . . . . . . . . . . . . . . . . . . . . . R . . . . . .   LYQK01000030.1
. . . . . . . . . . . . . G . . . . . . . . . . . . . . . . . . . . . . . . . . . . . R . . . . . .   CYUB01000012.1
. . . . . . . . . . . . . G . . . . . . . . . . . . . . . . . . . . . . . . . . . . . . . . . . . .   JAOYLY000000000
. . . . . . . . . . . . . G . . . . . . . . . . . . . . . . . . . . . . . . . . . . . R . . . . . .   JALCNG010000005
. . . . . . . . . . . . . G . . . . . . . . . . . . . . . . . . . . . . . . . . . . . R . . . . . .   CADILJ010000005
. . . . . . . . . . . . . G . . . . . . . . . . . . . . . . . . . . . . . . . . . . . R . . . . . .   CYTG01000002.1
. . . . . . . . . . . . . G . . . . . . . . . . . . . . . . . . . . . . . . . . . . . R . . . . . .   PHIH01000001.1
. . . . . . . . . . . . . G . . . . . . . . . . . . L . . . . . . . . . . . . . . . . R . . . . . .   JAQZZN010000001
. . . . . . . . . . . . . G . . . . . . . . . . . . . . . . . . . . . . . . . . . . . R . . . . . .   CP127192.1
. . . . . . . . . . . . . G . . . . . . . . . . . . . . . . . . . . . . . . . . . . . R . . . . . .   JABBZE000000000
. . . . . . . . . . . . . G . . . . . . . R . . . . . . . . . . . . . . . . . . . . . R . . . . . .   LVKO00000000.1
. . . . . . . . . . . . . G . . . . . . . . . . . . . . . . . . . . . . . . . . . . . R . . . . . .   CADIKY010000002
. . . . . . . . . . . . . . . . . . . . . . . . . . . . . . . . . . . . . . . . . . . . . . . . . .   WWES01000143.1
. . . . . . . . . . . . . G . . . . . . . . . . . . . . . . . . . . . . . . . . . . . R . . . . . .   LYQM01002536.1
. . . . . . . . . . . . . G . . . . . . . . . . . . . . . . . . . . . . . . . . . . . . . . . . . .   JAOYLZ000000000
. . . . . . . . . . . . . G . . . . . . . . . . . . . . . . . . . . . . . . . . . . . . . . . . . .   JAPZVH000000000
. . . . . . . . . . . . . G . . . . . . . . . . . . . . . . . . . . . . . . . . . . . . . . . . . .   JAOYMB000000000
. . . . . . . . . . . . . G . . . . . . . . . . . . L . . . . . . . . . . . . . . . . R . . . . . .   JAPZVL010000003
. . . . . . . . . . . . . G . . . . . . . . . . . . . . . . . . . . . . . . . . . . . R . . . . . .   CADIJL010000001
. . . . . . . . . . . . . G . . . . . . . . . . . . . . . . . . . . . . . . . . . . . . . . . . . .   JAKJWT010000002
. . . . . . . . . . . . . G . . . . . . . . . . . . . . . . . . . . . . . . . . . . . . . . . . . .   JAOYMA000000000

                  210                 220                 230                 240                 250
. . . . | . . . . | . . . . | . . . . | . . . . | . . . . | . . . . | . . . . | . . . . | . . . . |
D T L L F G D A L K P Q S R Q Q L Q S W L L A N T T G G K R L R A G M P A D W K I G E K T G T Y S K   LDVO01000240.1
. . . . . . . . . . . . . . . . . . . . . . . . . . . . . . . . . . . . . . . . . . . . . . . . . .   LDVL01000432.1
. . . . . . . . . . . . . . . . . . . . . . . . . . . . . . . . . . . . . . . . . . . . . . . . . .   JAJLPM010000175
. . . . . . . . . . . . . . . . . . . . . . . . . . . . . . . . . . . . . . . . . . . . . . . . . .   JAKJWO010000011
. . . . . . . . . . . . . . . . . . . . . . . . . . . . . . . . . . . . . . . . . . . . . . . . . .   LDVN01000442.1
. . . . . . . . . . . . . . . . . . . . . . . . . . . . . . . . . . . . . . . . . . . . . . . . . .   LDVK01000415.1
. . . . . . . . . . . . . . . . . . . . . . . . . . . . . . . . . . . . . . . . . . . . . . . . . .   JAJLPZ010000722
. . . . . . . . . . . . . . . . . . . . . . . . . . . . . . . . . . . . . . . . . . . . . . . . . .   JAKLXR010000011
. . . . . . . . . . . . . . . . . . . . . . . . . . . . . . . . . . . . . . . . . . . . . . . . . .   HE798385.1
. . . . . . . . . . . . . . . . . . . . . . . . . . . . . . . . . . . . . . . . . . . . . . . . . .   CP053618.1
. . . . . . . . . . . . . . . . . . . . . . . . . . . . . . . . . . . . . . . . . . . . . . . . . .   JAKLXU010000092
. . . . . . . . . . . . . . . . . . . . . . . . . . . . . . . . . . . . . . . . . . . . . . . . . .   CP025774.1
```

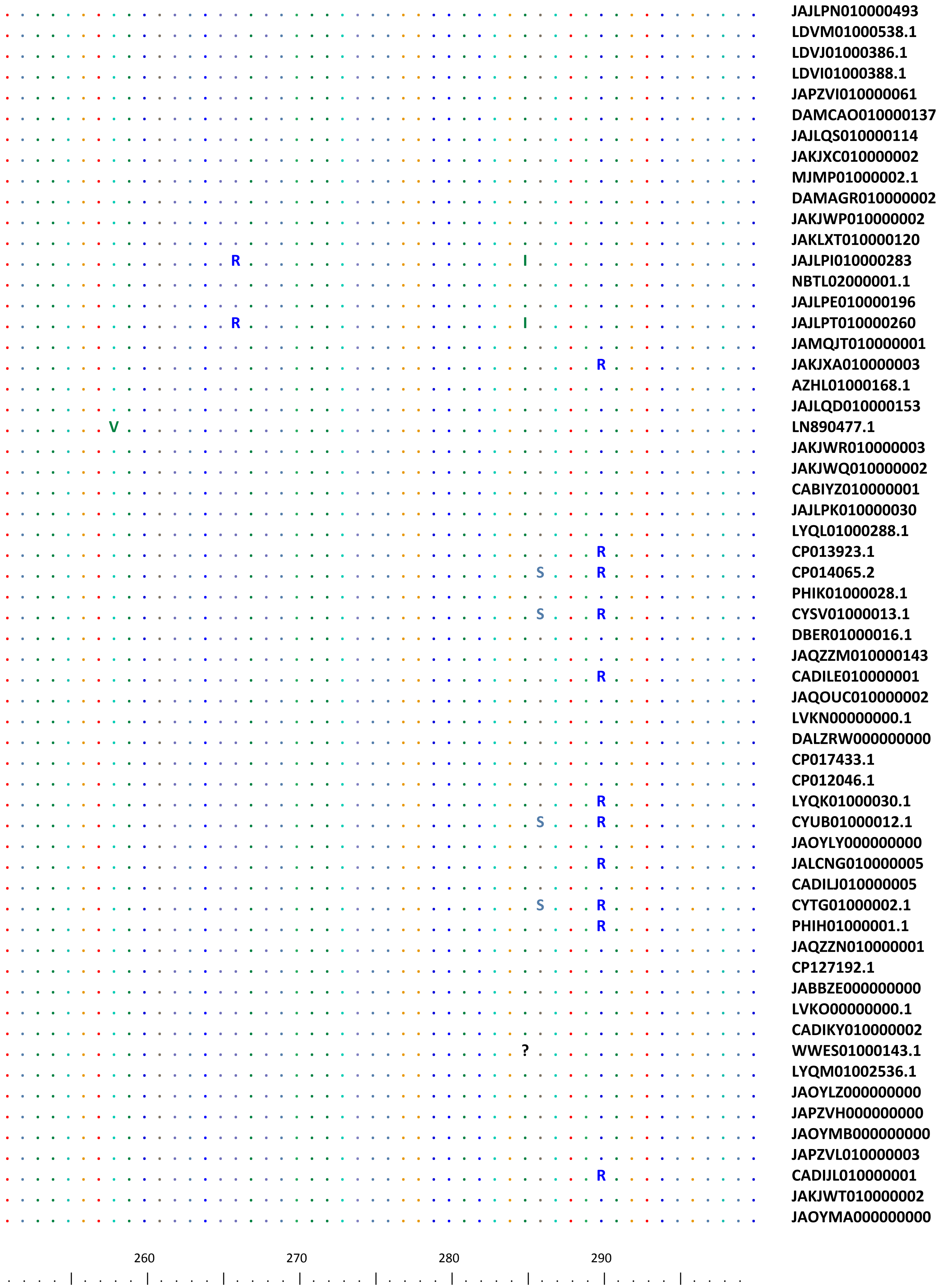
JAJLPN010000493
LDVM01000538.1
LDVJ01000386.1
LDVI01000388.1
JAPZVI010000061
DAMCAO010000137
JAJLQS010000114
JAKJXC010000002
MJMP01000002.1
DAMAGR010000002
JAKJWP010000002
JAKLXT010000120
R I JAJLPI010000283
NBTL02000001.1
JAJLPE010000196
R I JAJLPT010000260
JAMQJT010000001
R JAKJXA010000003
AZHL01000168.1
JAJLQD010000153
V LN890477.1
JAKJWR010000003
JAKJWQ010000002
CABIYZ010000001
JAJLPK010000030
LYQL01000288.1
R CP013923.1
S R CP014065.2
PHIK01000028.1
S R CYSV01000013.1
DBER01000016.1
JAQZZM010000143
R CADILE010000001
JAQOUC010000002
LVKN00000000.1
DALZRW000000000
CP017433.1
CP012046.1
R LYQK01000030.1
S R CYUB01000012.1
JAOYLY000000000
R JALCNG010000005
CADILJ010000005
S R CYTG01000002.1
R PHIH01000001.1
JAQZZN010000001
CP127192.1
JABBZE000000000
LVKO00000000.1
CADIKY010000002
? WWES01000143.1
LYQM01002536.1
JAOYLZ000000000
JAPZVH000000000
JAOYMB000000000
JAPZVL010000003
R CADIJL010000001
JAKJWT010000002
JAOYMA000000000
260
270
280
290

VGCNDAGFAQPPGAAPIIIAAYLETTAVPMEERDRCIAEVGRLVAALG	*	LDVO01000240.1
....	*	LDVL01000432.1
....	*	JAJLPM010000175
....	*	JAKJWO010000011
....	*	LDVN01000442.1
....	*	LDVK01000415.1
....	*	JAJLPZ010000722
....	*	JAKLXR010000011
....	*	HE798385.1
....	*	CP053618.1
....	*	JAKLXU010000092
....M.	*	CP025774.1
....	*	JAJLPN010000493
....	*	LDVM01000538.1
....	*	LDVJ01000386.1
....	*	LDVI01000388.1
..................................C.............	*	JAPZVI010000061
....M.	*	DAMCAO010000137
....	*	JAJLQS010000114
....	*	JAKJXC010000002
....	*	MJMP01000002.1
....	*	DAMAGR010000002
....	*	JAKJWP010000002
....	*	JAKLXT010000120
...................................A............	*	JAJLPI010000283
....	*	NBTL02000001.1
....	*	JAJLPE010000196
...................................A............	*	JAJLPT010000260
....	*	JAMQJT010000001
....	*	JAKJXA010000003
....	*	AZHL01000168.1
....	*	JAJLQD010000153
....	*	LN890477.1
....	*	JAKJWR010000003
....	*	JAKJWQ010000002
....	*	CABIYZ010000001
....	*	JAJLPK010000030
....	*	LYQL01000288.1
...........S....................................	*	CP013923.1
....	*	CP014065.2
....	*	PHIK01000028.1
....	*	CYSV01000013.1
....	*	DBER01000016.1
....	*	JAQZZM010000143
....	*	CADILE010000001
....	*	JAQOUC010000002
....	*	LVKN00000000.1
....	*	DALZRW000000000
....	?	CP017433.1
....	*	CP012046.1
....	*	LYQK01000030.1
....	*	CYUB01000012.1
....	*	JAOYLY000000000
....	*	JALCNG010000005
....	*	CADILJ010000005
....	*	CYTG01000002.1
....	*	PHIH01000001.1
....	*	JAQZZN010000001
....	?	CP127192.1
....	*	JABBZE000000000
....	*	LVKO00000000.1
....	*	CADIKY010000002

. . . . * WWES01000143.1

. . . . * LYQM01002536.1

. . . . * JAOYLZ000000000

. . . . * JAPZVH000000000

. . . . * JAOYMB000000000

. . . . * JAPZVL010000003

. . . . * CADIJL010000001

. . . . * JAKJWT010000002

. . . . R T C . - . . ? ? G G T . ? - - - - - - JAOYMA000000000

Figure S2: bla_{AXC} genetic context investigated in selected *A. xylosoxidans* and *A. ruhlandii* genomes downloaded from NCBI genome database. Clinker program was used for the alignment and graphing of the genetic context

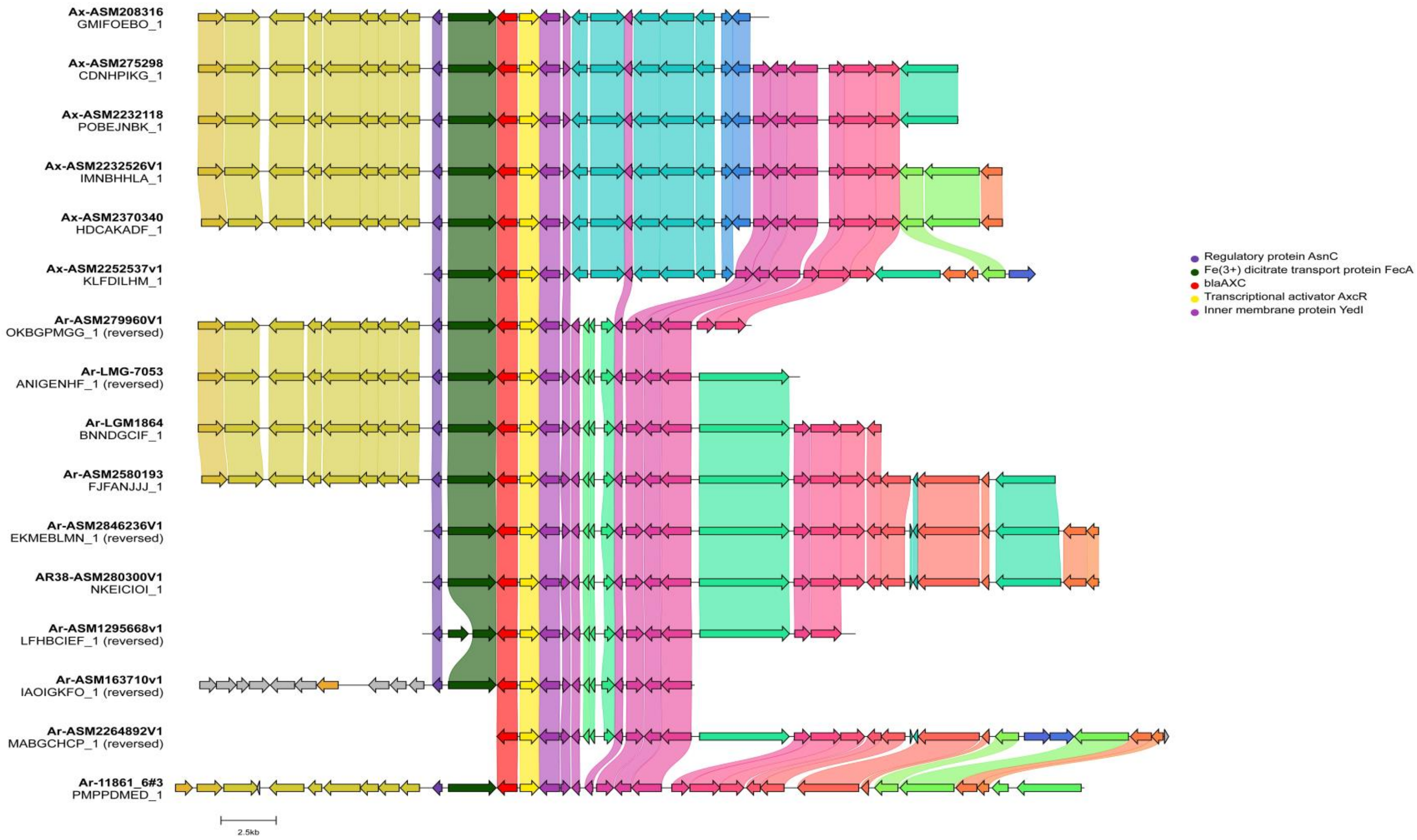

Supplement: Supplementary file 1 [file pathogens-13-00115-s001.zip › Fig S1 and S2 - Supplementary material-2.pdf]
